# Epilepsy and genetics

**DOI:** 10.1515/medgen-2022-2142

**Published:** 2022-09-22

**Authors:** Johannes R. Lemke

**Affiliations:** Institute of Human Genetics, University of Leipzig Medical Center, Leipzig, Germany; Center for Rare Diseases, University of Leipzig Medical Center, Leipzig, Germany

Epilepsy is one of the best-known and most prevalent disorders in humans [1]. The oldest (preserved) descriptions of epileptic seizures date back more than 4000 years [2]. Over the past millennia, the understanding of the etiology of epilepsy and thus its therapy has changed considerably. Babylonians assigned seizures to evil spirits and treated the condition through spiritual means [3]. The ancient Greeks associated epilepsy with genius and divine and therefore named it *Sacred Disease* [2]. At around 400 BC, Hippocrates appears to be the first who postulated a medically treatable disorder originating in the brain and even ascribed importance to heredity [2], [3]. Still, treatment of epileptic seizures remained spiritual and/or appalling for the following centuries and frequently appears absurd from a today’s perspective. It was not before the mid of the 19^th^ century when Bromide was introduced as the first evidence-based antiseizure medication (ASM) [4] followed by phenobarbital and phenytoin in the early 20^th^ century. Today, another century later, we are aware of a few dozen compounds being approved as ASM. However, as nowadays the individual etiology of epilepsy in an affected individual continues to remain vastly unknown, application of the various ASM still follow empirical recommendations mostly depending on the phenotype or sometimes solely on personal experience. The introduction of even more evidence-based approaches to treating epilepsy taking etiology into account appears to be most desirable.

This issue of *Medizinische Genetik* summarizes the current knowledge on epilepsy disorders and shed light on etiologic, diagnostic and treatment aspects.

The first manuscript “**Monogenic epilepsies and how to approach them in 2022**” (Krey et al., 1), we give an overview on how epilepsies are classified and what is known on the respective genetic contribution. Epilepsy can occur as isolated condition or be part of a more complex neurodevelopmental disorder. One quarter of people with epilepsy have intellectual disability and one fifth of people with intellectual disability have epilepsy [5]. The implementation of *Next Generation Sequencing* in routine genetic testing has not only revolutionized the field of epilepsy diagnostics. It becomes clear that standard genetic testing strategies comprising conventional techniques (such as karyotyping, chromosomal micro arrays, Sanger sequencing and also panel sequencing) should all be replaced by exome-first (or potentially even genome-first) approaches. This strategy is also recommended by the *International League Against Epilepsy* [6]. It is demonstrated that complex phenotypes will have the highest diagnostic yield. Thus, exome sequencing should particularly be considered in complex conditions (i. e. in developmental and epileptic encephalopathies).

In the second manuscript “**Genetic testing in adults with developmental and epileptic encephalopathy – What do we know?**” (Krey et al., 2), we focus on a striking diagnostic disparity between adults and children with epilepsy. Genetic epilepsies usually manifest in childhood and genetic testing is normally performed in proximity of the time of manifestation. Thus, affected children often receive comprehensive and up-to-date genetic testing, whereas adults normally have not undergone genetic testing as they did not have this opportunity in their childhood. However, only analysing adult individuals with neurodevelopmental disorders with epilepsy will enable the delineation of the longitudinal courses as well as late phenotypes and will deliver information urgently needed for genetic counselling of families with newly diagnosed children [7]. This disparity and the significant underrepresentation of adult and elderly individuals both in routine genetic diagnostic as well as research setting is particularly disadvantageous as little to nothing is known on respective long-term courses and late phenotypes. Genetic testing of adult individuals, will help understand the natural course of the disease and benefit newly diagnosed children and their relatives [7]. Moreover, in some cases it may even improve treatment and therapy of the respective adult individual itself – leading over to the third manuscript “**Developmental and epileptic encephalopathies – Therapeutic consequences of genetic testing**” (Syrbe) describing possibilities for precision medicine treatment arising from particular genetic diagnoses in the field of genetic epilepsies. Making the first steps into the era of precision medicine, we are now capable of making treatment decisions founded on the individual etiology of a respective condition in a single individual. Possibilities of precision medicine approaches comprise consideration of empiric knowledge as well as agonising/antagonising the molecular dysfunction, replacement therapies and even gene therapy. With these new opportunities at hand, we might be able to overcome the era of empirical phenotype-driven treatment recommendations and/or decision making depending on solely someone’s personal experience.

As already suggested by Hippocrates, we have proof today that genetic factors play a significant role in epileptogenesis – not only in monogenic but also in polygenic context. The fourth manuscript “**Polygenic risk scores in epilepsy**” (Heyne) addresses the large field of polygenic epilepsies that could thus far not be captured by routine genetic testing methods.

The implementation of polygenic risk scores in diagnostic settings will likely help stratifying individuals with seizures that cannot be assigned to monogenic etiologies. This approach will help to shed light on the yet vastly unexplored spectrum of complex epilepsies and has an enormous potential in changing our general perception of genetic testing in the future.
